# Antimicrobial peptides and proteins as rheostats of intestinal homeostasis and immunity

**DOI:** 10.1016/j.coi.2026.102738

**Published:** 2026-02-17

**Authors:** Talia Akoh-Arrey, John F Brooks

**Affiliations:** Princeton University, Department of Molecular Biology, United States

## Abstract

Antimicrobial peptides and proteins (AMPs) function as molecular rheostats of host–microbe interactions and cell-intrinsic defense. Rather than being binary effectors, they act along a continuum. At basal levels, AMPs maintain harmony with the commensal members of the microbiome, and upon pathogen encounter, they induce levels that thwart bacterial invasion and dissemination. This duality arises from the mechanistic versatility and layered regulation by microbial, cytokine, and circadian cues. Importantly, this review is not intended to be a detailed catalog of all antimicrobial proteins, but rather a conceptual framework highlighting representative mechanisms that illustrate how AMPs function as adjustable regulators of intestinal homeostasis. Viewing AMPs as adjustable regulators of barrier integrity, rather than static effectors, reframes innate immunity as a dynamic system that balances microbial tolerance with host defense.

## Introduction

### Antimicrobial proteins and peptides as evolutionary arbiters of mucosal immunity

The mammalian gastrointestinal tract negotiates a fundamental immunological paradox: defending against enteric pathogens while maintaining lifelong partnerships with trillions of commensal microorganisms. This balance is orchestrated in part by antimicrobial peptides and proteins (AMPs), evolutionary ancient effectors that function at the epithelial–microbial interface across metazoans [1–4]. Rather than functioning as simple on-off switches, AMPs act as molecular rheostats that tune microbial load, composition, and spatial organization [5–10].

**Figure 1:**
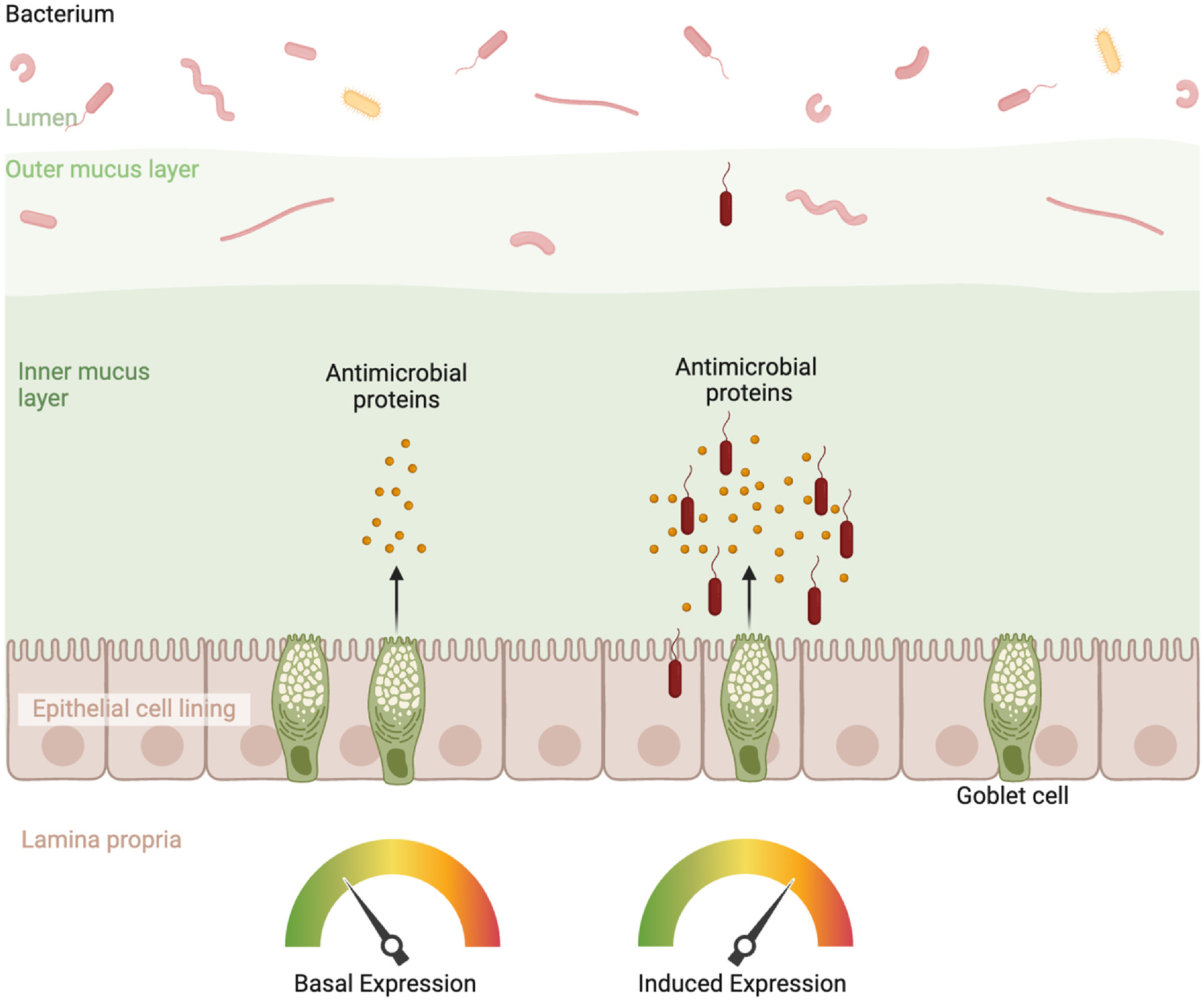
AMPs as molecular rheostats of intestinal barrier maintenance. Conceptual model depicting AMP concentration-dependent effects. Basal-level AMP expression (maintained by TLR/cytokine signaling and circadian rhythms) enforces spatial segregation and maintenance of commensal populations, whereas high induction of AMP expression (triggered by pathogen encounter and amplified through PRR signaling circuits) enables pathogen clearance. This rheostat function is tuned by microbial density, epithelial-immune crosstalk, metabolic state, and circadian phase. Dysregulation through genetic deficiency, circadian disruption, or dietary perturbation results in barrier failure or dysbiosis. Figure was created with BioRender.com.

Host-associated microbial communities have co-evolved intricate partnerships with their animal hosts, calibrating metabolism and immunity in ways that promote mutual benefit [11–15]. AMPs enable this symbiosis through dual functionality: at low production, they foster spatial segregation and symbiont persistence, and at high production, enable pathogen clearance (Figure 1). This is most evident by the induction of select AMPs during enteric infection [6,16]. By conceptualizing AMPs as rheostats rather than binary effectors, we reveal a unifying logic linking molecular mechanisms to microbial ecology. Here, we synthesize a current understanding of how mammalian intestinal AMPs execute this rheostat function, focusing on mechanistic strategies, regulatory circuits, and unresolved questions central to host–microbe interactions.

### Antimicrobial peptide and protein mechanisms as a functional spectrum

Rather than discrete categories, bactericidal and non-bactericidal AMPs represent a functional continuum of biochemical strategies that collectively maintain barrier integrity. This spectrum-based view accommodates both host-centric regulatory logic and microbial adaptive responses, which together determine whether AMP exposure results in tolerance, spatial exclusion, or elimination. Together, these mechanisms reveal a simple principle: the intestinal epithelium deploys multiple solutions to constrain microbial encroachment while preserving key symbiotic partners. This mechanistic diversity raises fundamental questions about evolutionary convergence and functional specialization. Why have mammals evolved such varied antimicrobial arsenals? The answer likely lies in the complexity of the microbial challenge itself: diverse bacterial species with distinct cell morphology, variable growth states, and adaptive resistance mechanisms require correspondingly diverse countermeasures. Moreover, different anatomical niches, from the sparingly colonized small intestine to the densely colonized large intestine, impose distinct selective pressures that seemingly favor specialized AMP repertoires [17].

### Lysozyme: working at the cell wall

Lysozyme is a bacteriolytic enzyme and among the most abundant AMPs produced within the intestine [6].

This enzyme targets the rigid meshwork of peptidoglycan that comprises the bacterial cell wall and hydrolyzes the glycosidic bonds between the alternating subunits of peptidoglycan, resulting in cell lysis [6,18]. Under homeostatic conditions, lysozyme is secreted in coordination with other AMPs. However, in the context of *Salmonella* infection, large payloads of lysozyme are mobilized through a defined pathway known as secretory autophagy to prevent bacterial dissemination [6]. The secretory autophagy pathway enables rapid, high-volume lysozyme secretion during infection without the necessity of new transcription. This context-dependent mobilization of lysozyme, as both a constitutive barrier component and an inducible pathogen defense factor, embodies the rheostat concept at the level of a single protein.

### Membrane-disrupting antimicrobial peptides and proteins: exploiting charge asymmetry

The predominant bactericidal strategy exploits membrane charge asymmetry among bacterial and host cell membranes. The distinction between anionic bacterial membranes versus neutral eukaryotic membranes enables selective targeting by key antimicrobial proteins. REG3γ, RELMβ, α-defensins, sPLA2-IIa, and SPRR2A all leverage this principle through distinct mechanisms [16,19–28]. The cationic effectors REG3γ, RELMβ, and the α-defensins form hexameric, multimeric, and dimeric pores, respectively [28–30]. sPLA2-IIa promotes membrane disruption by hydrolyzing anionic phospholipids [25–27]. SPRR2A harbors a series of cysteines that form high-order disulfide bonds [16]. This disulfide bond formation is critical for the binding of the negatively charged lipids and subsequent bactericidal activity [16]. However, the specific mechanism by which SPRR2A promotes membrane disruption is currently unknown. This convergence of charge-based membrane disruption reflects strong selectivity against targeting of the host cell membrane. Interestingly, these AMPs are partitioned among those that selectively target Gram-positive bacteria (REG3γ, sPLA2-IIa, and SPRR2A), those that target Gram-negative bacteria (RELMβ), and α-defensins, which can target both. Mechanistically, lipopolysaccharide (LPS) present on the outer membranes of Gram-negative bacteria inhibits the biochemical activity of REG3γ, sPLA2-IIa, SPRR2A, and even lysozyme [5,16,20,26,31]. Interestingly, Peptide YY (PYY) extends membrane-disrupting AMP function beyond bacteria to fungi, selectively killing the hyphal (pathogenic) form of *Candida albicans* while sparing the yeast (commensal) form [32]. This morphotype selectivity prevents fungal pathogenesis while preserving fungal commensalism through membrane permeabilization, illustrating how AMPs discriminate between benign and harmful states of the same organism, a rheostat function operating at the level of microbial morphology. The convergence of function, coupled with the diversity of targets, ensures broad antimicrobial activity at the intestinal barrier, denoting cooperative antimicrobial networks.

### Agglutinating and sequestering antimicrobial peptides and proteins: spatial control without killing

Non-bactericidal AMPs maintain the intestinal barrier through restriction rather than elimination. Lypd8 selectively binds bacterial flagella and restricts motility, preventing flagellated bacteria from traversing the mucus layer [33,34]. ZG16 agglutinates bacteria by binding N-acetylmuramic acid in peptidoglycan, preventing colonization of the inner mucus layer [35,36]. From a microbial perspective, these mechanisms impose selective pressure favoring spatial avoidance strategies rather than outright resistance, enabling commensals to persist by occupying permissive niches rather than confronting stress imposed by antimicrobial proteins directly. Like SPRR2A and REG3γ, both proteins are inhibited by LPS, conferring Gram-positive selectivity [35,36]. Together, Lypd8 and ZG16 establish spatial boundaries that segregate microbes from the intestinal epithelium without overt elimination.

### Metabolic and immunomodulatory antimicrobial peptides and proteins: indirect antimicrobial control

Recent discoveries reveal that some AMPs function through metabolic coupling or immune signaling rather than direct antimicrobial action, expanding the definition of ‘antimicrobial’ beyond killing. Mouse Apol9a/b, produced by enterocytes, preferentially coats commensal bacteria and induces outer membrane vesicle (OMV) formation [37]. These OMVs enhance interferon-γ signaling in intraepithelial lymphocytes and promote MHC class II expression. Strikingly, Apol9a/b activity requires commensal-derived ceramide-1-phosphate lipids, demonstrating bidirectional metabolic communication in which symbionts modulate host defense programs [37].

Rather than passive targets, commensals emerge as active participants requiring metabolically demanding ceramide-1-phosphate synthesis to engage Apol9a/b, highlighting a molecular dialog linking AMP sensitivity to symbiotic competence [37]. This creates a positive feedback loop in which metabolically competent symbionts enhance barrier immunity, potentially excluding metabolically deficient pathogens that cannot trigger Apol9a/b-mediated protection. A human APOL2 ortholog likewise coats commensal bacteria to liberate OMVs, whereas human APOL3 is directly bactericidal to enteric pathogens invading host cells [38]. Whether other AMPs similarly depend on commensal-derived cofactors remains unexplored but represents a promising avenue for understanding symbiotic maintenance.

### Antimicrobial peptide and protein cooperation: synergism and functional redundancy

Emerging evidence suggests that AMPs function cooperatively rather than independently, creating antimicrobial networks whose activity exceeds the sum of individual components. For example, sPLA2-IIa’s ability to target Gram-negative bacteria when combined with membrane-permeabilizing factors exemplifies this synergism: lipid hydrolysis may destabilize outer membranes, enabling access for pore-forming lectins like REG3γ [24–26]. Similarly, lysozyme’s hydrolysis of bacterial cell components may allow for access to the inner cell membrane by pore-forming AMPs, such as α-defensins.

Whether such coordination is actively regulated or emerges from overlapping expression patterns remains unclear, but the principle suggests that AMPs should be studied as integrated systems rather than isolated effectors. Preliminary evidence hints at hierarchical deployment: constitutive AMPs like lysozyme may provide baseline barrier maintenance, while inducible AMPs like REG3γ and SPRR2A amplify responses during infection or helminth challenge. We speculate that this temporal layering could minimize resistance evolution by exposing bacteria to fluctuating immune pressure rather than constant antimicrobial environments. This is further exemplified by the rhythmic production of AMPs, like REG3γ, for which a controlled burst of activity throughout the day-night cycle may promote barrier maintenance while potentially minimizing the development of microbial resistance [39–41]. This hypothesis is supported by *in vitro* studies in which temporal pulsing of antibiotics decreased selective pressure as quantified by resistance development based solely on the duration of the pulse and the adaptability of the bacteria to fluctuating stresses within the environment [42].

Functional redundancy among AMPs may represent an evolutionary safeguard against resistance. When pathogens acquire resistance to one AMP class (e.g. membrane-disrupting peptides), alternative mechanisms such as agglutination or motility restriction remain effective. At first glance, this functional redundancy appears to conflict with the mechanistic specialization observed among AMP families. However, these concepts can be reconciled by distinguishing functional redundancy (overlapping ecological outcomes such as spatial exclusion or growth limitation) from mechanistic specialization (distinct biochemical pathways achieving those outcomes). Under homeostatic conditions, mechanistic specialization likely dominates; however, during infection or breach of the barrier, redundancy becomes advantageous, ensuring robustness against pathogen adaptation or AMP resistance. This balance between specialization and redundancy is further shaped by defined regulatory signaling pathways, allowing AMP networks to be precisely tuned in both space and time, as discussed in more detail below. Thus, redundancy and specialization are not opposing principles but context-dependent features of an adaptive antimicrobial network.

### Regulatory networks integrating microbial and host signals

AMP expression is dynamically regulated through overlapping pattern recognition receptor (PRR) pathways, cytokine circuits, and circadian rhythms. In addition to canonical TLR-MYD88 signaling, alternative epithelial signaling pathways also converge on AMP regulation. Notably, TNF superfamily receptor 14 (HVEM) signaling in intestinal epithelial cells (IECs) promotes REG3γ expression, highlighting that multiple immune receptor systems independently reinforce antimicrobial barrier programs [43]. Rather than responding to individual signals, the intestinal epithelium integrates multiple inputs to calibrate AMP output to microbial context, a logic that enables context-appropriate responses.

### TLR-MYD88-ILC3-IL-22 axis: a canonical regulatory circuit

Host cells detect microbial molecular patterns through a network of PRRs, among which Toll-like receptors (TLRs) are the most extensively characterized. These transmembrane receptors recognize conserved microbial ligands and couple ligand binding to downstream transcriptional programs that induce AMP production and intestinal barrier fortification [44–46].

TLR signaling relevant to AMP induction operates in both IECs and sub-epithelial myeloid cells [47,48]. Previous work has determined a role for TLR signaling specifically within epithelial cells as required for REG3γ expression [49]. In addition to epithelial cell-intrinsic regulation, a role for myeloid cell TLR-dependent signaling has also been observed in the regulation of REG3γ [47–49]. Specifically, TLR engagement in myeloid cells within the sub-epithelial space stimulates IL-23 release, which acts on Group 3 Innate Lymphoid cells (ILC3s) to trigger IL-22 production [47,48]. IL-22 then binds its receptor on epithelial cells, activating the transcription factor signal transducer and activator of transcription (STAT3), which drives expression of REG3γ [40,47,48].

Strikingly, colonization with segmented filamentous bacteria (SFB), a Gram-positive commensal that adheres to the epithelium, is sufficient to activate this myeloid-ILC3-IL-22 axis, demonstrating that bacterial attachment, not just microbial presence, is a key determinant of AMP induction [37,40,47,48]. In addition to REG3γ, RELMβ expression is also induced by the cytokine IL-22, as treatment of organoids with IL-22 results in the upregulation of RELMB [50]. Finally, Type II cytokines (IL-4 and IL-13) activate STAT6 to induce SPRR2A and RELMβ during helminth infection [16], while IFN-γ upregulates REG3γ, RELMβ, and Lypd8 [50]. This regulatory redundancy ensures robust AMP responses across diverse infectious contexts.

Most striking is the coordination of key responses across the AMP network. During helminth infections, which promote a Type II cytokine response, SPRR2A and RELMβ expression is induced, while the expression of REG3γ, lysozyme, and the α-defensins is diminished [16]. Although this induction may simply be due to the expansion of Goblet cells, the cell type that produces these AMPs, in the presence of Type II cytokines, the functional consequence of altering the temporal pattern of AMP expression remains unclear.

### Spatial-temporal regulation of the intestinal barrier: tuning the rheostat

AMP expression exhibits striking regional specialization. REG3γ, lysozyme, α-defensins, sPLA2-IIa, and PYY dominate the small intestine, while RELMβ, Lypd8, and ZG16 are enriched in the large intestine [16,27,32–36,49]. This spatial patterning persists despite the broad distribution of upstream regulatory pathways (e.g. IL-22/STAT3, TLR-MYD88), suggesting that regional AMP expression is determined by microbial biogeography and localized immune populations. ILC2s and IL-13 are concentrated in the proximal colon, driving RELMβ [17], while SFB and IL-22-producing ILC3s localize to the distal ileum, promoting REG3γ [5,37,40,47]. Although the physiological consequences of this regionalization have yet to be defined, future work mapping spatiotemporal AMP expression at high resolution may reveal ordered deployment strategies that maximize barrier protection while minimizing resistance selection.

Yet spatial patterning alone cannot explain the precision of AMP deployment. The small intestine harbors relatively low bacterial density but high nutrient availability and rapid transit, favoring bactericidal AMPs that prevent pathogen colonization during the brief window of nutrient exposure. The colon, by contrast, harbors dense bacterial communities with slower transit, favoring spatial segregation strategies (Lypd8, ZG16) that maintain physical separation rather than attempting wholesale bacterial elimination. This anatomical specialization suggests that AMP selection pressure varies with local ecological constraints.

Emerging evidence indicates that AMP regulation is also temporally controlled. REG3γ expression oscillates with circadian rhythms, coordinated by the microbiota and the host circadian clock [39,40,50]. These rhythms synchronize with bacterial activity cycles: REG3γ peaks during active feeding periods when bacterial loads surge, then declines during fasting [39,40,50]. A high-fat diet disrupts these rhythms, leading to dysregulated REG3γ expression and microbiota composition changes that promote metabolic dysfunction [39,40,50]. Feeding-dependent VIP neuron–ILC3 circuits further link nutrient sensing to barrier regulation [41], suggesting that metabolic state directly tunes antimicrobial pressure.

This temporal regulation may serve multiple functions. Here, we propose several testable predictions that suggest circadian regulation of innate immune functions may shape host–microbe interactions. First, rhythmic AMP oscillations may prevent resistance evolution by exposing bacteria to fluctuating barrier pressure, a strategy analogous to pulsed antibiotic therapy. Second, synchronized AMP induction during feeding may coincide with peak pathogen exposure, maximizing protection when vulnerability is highest. Third, circadian AMP cycling may coordinate with systemic physiology, linking glucose metabolism and infection susceptibility to time-of-day-dependent immune competence. Supporting this model, studies have reported that mice infected at different circadian phases exhibit variable mortality, with infections during the sleep phase (when REG3γ is low) proving more lethal [40]. Finally, rhythmic pulsing may promote protection of mucosal surfaces while retaining key microbial partners, as overproduction of AMPs has deleterious consequences for microbial maintenance [51]. Testing these hypotheses requires longitudinal monitoring of AMP concentrations, microbial activity, and infection outcomes across circadian cycles.

### Synthesis: the antimicrobial peptide and protein rheostat model

Integrating these regulatory mechanisms reveals how AMPs function as molecular rheostats. At homeostasis, AMP expression is maintained by basal TLR and cytokine signaling, enforcing spatial segregation without eliminating commensals. Upon pathogen invasion or barrier breach (signaled by increased PRR activation and cytokine release), AMP expression, production, and deployment are amplified through coordinated epithelial-immune signaling circuits. This inducible response clears pathogens while generating transient antimicrobial pressure that potentially prevents resistance evolution among commensals. As infection resolves, basal feedback loops prevail and retract AMP expression to homeostatic levels, restoring symbiotic balance. This dynamic regulation, tuned by microbial density, immune status, metabolic state, and circadian phase, enables AMP production to correspond with the potential threat level and mobilizes barrier defense without requiring slower adaptive immune responses.

## Future directions

### How does the host maintain antimicrobial peptide and protein sensitivity among commensals?

Loss of key AMPs often promotes bacterial encroachment of the intestinal epithelium and enhances pathogen dissemination, indicating that neither enteric pathogens nor commensals are uniquely resistant to endogenous AMPs. Therefore, a central paradox remains unresolved: how do commensal bacteria persist despite continuous AMP exposure? This paradox highlights microbial agency. Commensals are not passive targets but active participants capable of sensing, adapting to, and exploiting fluctuating antimicrobial landscapes. Below, we delineate four hypotheses.

First, coevolutionary tuning may have selected commensal strains with intrinsic sensitivity that promotes symbiosis, much as Apol9a/b activity depends on commensal-derived ceramide-1-phosphate [37]. This model predicts that commensals actively maintain AMP sensitivity through conserved biosynthetic pathways, with resistance mutations incurring fitness costs that reduce competitiveness. This mechanism has been observed with new classes of antibiotics that exhibit species selectivity [52]. Second, spatial segregation enforced by AMPs like Lypd8 and ZG16 may limit exposure while maintaining barrier protection. Commensals residing in the outer mucus layer or luminal space experience lower AMP concentrations than those attempting epithelial invasion, creating a concentration gradient that permits colonization while preventing breaching. Third, metabolic coupling may create feedback loops that prevent resistance evolution. Resistant strains may lose the ability to produce molecules such as ceramide-1-phosphate that enable beneficial host–microbe interactions, effectively linking AMP sensitivity to symbiotic competence. Fourth, the adaptive immune system may selectively clear resistant clones through mechanisms that preferentially target bacteria breaching AMP-mediated spatial boundaries. IgA coating, for instance, concentrates on bacteria invading the inner mucus layer, precisely where AMP pressure is highest. This suggests that adaptive and innate immunity cooperate to eliminate resistant mutants before they can establish. Finally, other potential strategies include inducible stress responses, envelope remodeling, metabolic-state-dependent sensitivity, and spatial repositioning within the mucus layer. Notably, these adaptations need not confer classical resistance; instead, they may enable commensals to remain below activation thresholds of inducible host defenses.

Distinguishing among these mechanisms requires longitudinal tracking of microbial evolution in AMP-deficient and AMP-competent hosts, coupled with analysis of spatial positioning and metabolic activity. Experimental evolution studies in which commensals are passaged through AMP-deficient mice, then reintroduced to AMP-competent hosts, could reveal whether AMP sensitivity is actively maintained by selection or passively retained due to lack of resistance mutations. These goals could also be achieved via experimental evolution strategies that involve bacterial growth in the presence of key AMPs, outside the host, and reintroduction of the evolved bacteria into AMP-competent animals. Gnotobiotic systems enabling controlled AMP dosing would further test whether commensals exhibit fitness costs upon acquiring resistance.

### Does the circadian clock play a role in synchronizing antimicrobial peptide and protein expression with environmental exposure?

Recent studies suggest that spatial and temporal regulation of AMP expression may function to minimize resistance evolution while enabling rapid pathogen clearance [17,39–41]. This raises a provocative model: AMPs may function as inducible rheostats that amplify during infection and retract during homeostasis, balancing pathogen elimination with commensal preservation. Do other AMPs, beyond the one described, exhibit phasic expression patterns with characteristic rise and fall kinetics? Are these patterns coordinated by feeding, circadian rhythms, or microbial feedback? How do pathogens and commensals differentially respond to transient versus sustained AMP exposure? Addressing these questions may reveal that temporal patterning, not absolute AMP levels, determines host–microbe outcomes. Inducible AMP knock-in systems with tunable expression kinetics would enable precise testing of dose–-duration relationships, while closed-loop bioreactors modeling pulsed AMP exposure could identify resistance-minimizing temporal patterns.

## Conclusion

Antimicrobial proteins and peptides are not merely defensive weapons during cell-intrinsic immunity but essential architects of intestinal symbiosis. By functioning as rheostats rather than switches, AMPs integrate host regulatory logic with microbial adaptive capacity, enabling coexistence rather than sterilization. Loss of individual AMPs consistently disrupts barrier integrity, underscoring their collective indispensability. Yet fundamental questions remain: How do commensals evade eradication by AMPs? Do temporal dynamics prevent resistance? Answering these questions will require moving beyond cataloging individual AMP mechanisms toward understanding their integrated function as a dynamic regulatory network. AMPs thus represent a universal strategy through which hosts convert innate immunity into a temporally and spatially dynamic language of coexistence.
